# Outcome of intracytoplasmic sperm injection after preinstillation of a gonadotropin releasing hormone agonist in the uterine cavity just before embryo transfer

**DOI:** 10.4274/tjod.23540

**Published:** 2017-06-15

**Authors:** Pınar Çağlar Aytaç, Bülent Haydardedeoğlu, Halis Özdemir, Esra Bulgan Kılıçdağ

**Affiliations:** 1 Başkent University Faculty of Medicine, Department of Obstetrics and Gynecology, Division of Reproductive Endocrinology and Infertility, Adana, Turkey; 2 Başkent University Faculty of Medicine, Department of Obstetrics and Gynecology, Division of Perinatology, Adana, Turkey

**Keywords:** Gonadotropin releasing hormone agonist, embryo transfer, uterine cavity, in vitro fertilization

## Abstract

**Objective:**

To evaluate the effects of a gonadotropin releasing hormone agonist (GnRHa) injection prior to embryo transfer on implantation and pregnancy rate.

**Materials and Methods:**

We performed a retrospective analysis of patients undergoing in vitro fertilization (IVF) therapy with and without GnRHa preinstallation into the uterine cavity just before embryo transfer between January 2012 and March 2013 in a single IVF center of a university hospital. Patients were evaluated based upon implantation, pregnancy, live birth, and miscarriage rates.

**Results:**

GnRHa was injected into the uterine cavity of 108 patients prior to embryo transfer which were regarded as study group. One thousand forty-seven patients who were not injected GnRHa were regarded as the control group. Pregnancy rates were 44.4% and 41.7% in the GnRHa and control groups, respectively. Live birth rates were 27.8% and 26.1%, miscarriage rates were 15.7% and 15.7%, and implantation rates were 31% and 30%, respectively and there were no difference between groups statistically (p>0.05).

**Conclusion:**

No statistically significant differences in implantation, pregnancy, live birth, or miscarriage rates were observed in patients treated with GnRHa prior to embryo transfer, relative to the controls. Therefore, GnRHa injection into the uterine cavity prior to embryo transfer is not recommended as a means of increasing implantation or pregnancy rates in IVF. However, prospective randomized controlled studies are needed to clarify the effect of GnRHa instillation in the uterine cavity for embryo implantation in IVF.

## PRECIS:

Gonadotropin releasing hormone agonist instillation into the uterine cavity before embryo transfer is not recommended to increase implantation and pregnancy rate in *in vitro* fertilization.

## INTRODUCTION

The success of in vitro fertilization (IVF) as a means of achieving clinical pregnancy is primarily dependent on the presence of high quality embryos and a suitable endometrium for implantation. Although the definition of high quality embryos is well established based upon morphologic criteria of the cleavage embryo or blastocyst, there remains no widely accepted morphologic or structural criterion that is used to assess the suitability of the endometrium. Therefore, much of the research in IVF has been focused on improving implantation rates through processes such as luteal phase support, which is now strongly recommended in IVF^(1)^.

Progesterone, estradiol, human chorionic gonadotropin (hCG), or gonadotropin releasing hormone agonists (GnRHa) have been evaluated in a variety of clinical studies as a means of luteal phase support to increase implantation and pregnancy rates in IVF^(2,3,4,5)^. More recent studies have suggested that intrauterine injections of hCG just before embryo transfer (ET) can increase pregnancy rates^(2,6,7)^. In cases of thin endometrium and recurrent pregnancy loss, injection of filgrastim (granulocyte colony-stimulating factor) into the uterine cavity is suggested as a means of increasing implantation and pregnancy rates^(8,9)^.

It has been hypothesized that the local expression of GnRH and its receptor may play a role in endometrial conditioning during implantation because GnRH receptors are present in the follicles, embryo, placenta, tubal epithelium, and endometrium^(10,11,12,13,14)^. Similarly, Casan et al.^(12)^ hypothesized that GnRH receptors in the tubal epithelium may play a role in fertilization, early embryonic development, and implantation during the early luteal phase via a combination of paracrine and autocrine methods. Here, we compared the effects of GnRHa injected directly into the uterine cavity prior to ET on implantation rate, pregnancy, and live birth rates. This is the first study in the literature of GnRHa pre-installation before ET to investigate improvements in implantation and pregnancy rates in IVF.

## MATERIALS AND METHODS

In our study, we evaluated the effects of GnRHa injection prior to ET on implantation and pregnancy rates in our IVF programme executed between January 2012 and March 2013. Outcomes of IVF cycles during that period were collected retrospectively. Injection of GnRHa into the uterus before ET was a standard treatment protocol used by a single physician (BH) in our clinic during that period. Outcomes of IVF cycles treated with GnRHa were compared with those that received IVF treatment without GnRHa. Informed consent was obtained from all patients prior to the initiation of any procedure. This study was approved by the Institutional Review Board of Başkent University, Ankara, Turkey (approval number: KA 15/302).

Only fresh cycles in which ET was performed were included. In addition, only the cycles of patients who were aged ≤40 years at the time of treatment, whose embryos were grade 1 or 2, and whose endometrial thickness was ≥7 mm on the day of hCG were included. The exclusion criteria included cycles comprising difficult ET, cancellations for other reasons, and patients who had 3 or more previous unsuccessful IVF cycles. A total of 1965 consecutive IVF cycles were performed in our clinic between January 2012 and March 2013, among which 142 cycles received GnRHa injection before ET. From this cohort of IVF cycles, 1155 met the necessary inclusion criteria, including 108 who received GnRHa injection before ET and 1047 that did not. Primary outcomes in the two groups were implantation and live birth rates, and secondary outcomes were pregnancy and miscarriage rates.

### Ovarian hyperstimulation and oocyte pick up

Ovarian hyperstimulation was performed according to the long protocol, antagonist protocol, or clomiphene citrate-follicle stimulating hormone (CC-FSH) protocol. In the long protocol, downregulation was achieved using GnRH analogues initiated at the start of the proceeding luteal phase, with dosing halved beginning on the day of hyperstimulation and continued until the day of hCG treatment. The patients were then treated with recombinant FSH in the early follicular phase. In the antagonist protocol, FSH analogues were started at the beginning of the follicular phase of the cycle, and GnRH antagonists were added when the leading follicles reached 12-13 mm in diameter. In the CC-FSH protocol, patients were treated with 50 mg clomiphene citrate twice a day beginning on the third day of the cycle, and augmented with FSH on the third day of CC treatment. In all protocols, ovulation was induced using 250 µg recombinant hCG that was administered after the leading follicle reached a minimum of 17 mm in diameter.

Oocyte retrieval was performed at 32-34 h after hCG administration. Oocytes were collected transvaginally. Metaphase II (MII) oocytes were prepared for the intracytoplasmic sperm injection (ICSI) procedure, which was performed after 2-2.5 h incubation. Embryos were transferred at the cleavage stage (3 days after ICSI).

### Embryo grading

Embryos were graded according to the number and equivalence of cells and the percentage of vacuoles formed around them, the presence of multinucleation, and the status of the zona pellucida on the second and third days. According to the morphologic criteria above, embryos were graded as 1-4 on the cleavage stage embryo. According to Turkish legal regulations, patients aged younger than 35 years can only receive one embryo, and two embryos can be transferred in patients aged 35 years or older, as well as in patients who have already undergone two unsuccessful ICSI cycles. Thus, one or two embryos were transferred on the third day after fertilization.

### Gonadotropin releasing hormone agonist injection and embryo transfer

For patients receiving GnRHa treatment, the vaginal cavity was washed with warm saline and embryo medium, followed by treatment with 0.5 mL GnRHa (including 0.1 mg/mL triptoreline acetate, Ferring, Greece), which was delivered via an embryo catheter under abdominal ultrasonography guidance. GnRHa was injected into the uterine cavity when the tip of the catheter reached 1 cm below the inner cavity of uterine fundus. Pre-selected embryos were then transferred into the uterine cavity after 10 min.

### Luteal support

All patients received luteal support, including 90 mg/day progesterone administered intra-vaginally starting on the day of ET and continued for 12 days.

Serum hCG levels were analyzed on day 12, with levels >10 mIU/mL suggestive of pregnancy. Clinical pregnancy was accepted as positive if the fetal gestational sac was apparent and a fetal heartbeat was regularly detected. The implantation rate was calculated as the number of gestational sacs seen on transvaginal ultrasound per number of transferred embryos. Miscarriage rates were calculated as the number of miscarriages before 12 weeks of gestation per cycles of ET. The live birth rate was calculated as the number of live births per IVF cycles with ET.

### Statistical Analysis

Demographic data for each group are presented as the mean ± standard deviations, or the median, with minimum and maximum or percentage values. The chi-square test was used to compare categorical data, and the Mann-Whitney U test was used to compare continuous variables that were non-normally distributed. Student’s t-test was used to compare continuous data with normal distribution. P values <0.05 were considered statistically significant. Data were analyzed using SPSS version 18.0 for Windows (SPSS, Inc., USA).

## RESULTS

After the exclusion of 34 cycles from the study and 474 cycles from the control groups, 108 IVF cycles formed the study and 1047 IVF cycles formed the control groups. Thirty-four cycles in the GnRHa group were excluded from the study.

Patient characteristics including age, duration of infertility, bilateral ovarian antral follicle counts on the day of early follicular phase, estradiol and progesterone levels, endometrial thickness on the day of hCG injection, duration of ovarian hyperstimulation, total dose of gonadotropins used for ovarian stimulation, number of total oocytes and MII oocytes obtained on the oocyte pick-up day, grade of the embryos, number of transferred embryos, and the reasons of IVF cycles were similar in both groups (Table 1).

Ovarian hyperstimulation was achieved using the long, agonist, or CC-FSH protocols. The long protocol was used in 23.1% and 25% of cycles for the GnRHa and controls groups, respectively. The agonist protocol was used for 74.1% and 70.6% of cycles, and the CC-FSH protocol was used in 4.4% and 2.8% of cycles in the GnRHa and control groups, respectively (p=0.63).

Pregnancy, live birth, miscarriage, and implantation rates were 44.4%, 27.8%, 15.7%, and 31%, respectively, in the GnRHa group compared with 41.7%, 26.1%, 15.7%, and 30%, respectively, in the control group. No statistically significant differences were observed between the two groups in terms of IVF results (Table 2). Similarly, the transfer of one or two embryos did not affect pregnancy, live birth, or implantation rates in the GnRHa group relative to the controls (p>0.05) (Table 3).

## DISCUSSION

In this study, we compared pregnancy-related IVF outcomes in patients treated with GnRHa immediately before ET with those receiving similar treatment without GnRHa stimulation. We observed no statistically significant differences in terms of implantation, pregnancy, live birth, or miscarriage rates between the groups.

During IVF treatment, spontaneous pregnancy is encountered in 1% of cycles using the long protocol when GnRHa is started during the previous luteal phase, consistent with an observation that inadvertent GnRHa injection might improve pregnancy rates^(15)^. Luteal phase injection of GnRHa has been suggested as a method to increase pregnancy and live birth rates by enhancing luteinizing hormone secretion and supporting maintenance of the corpus luteum^(5,16,17)^. Alternatively, GnRHa is also thought to affect GnRH receptors in the endometrium and embryo, thereby increasing implantation and pregnancy rates in donor cycles^(5)^. Based upon these observations, GnRHa injection directly into the uterine cavity has been suggested as a possible method for enhancing implantation rate, although no direct analyses have been performed.

Previous studies have offered strong support for the use of intrauterine therapy as a means of improving IVF outcomes. In a series of prospective, randomized controlled studies, hCG injection into the uterine cavity before ET increased pregnancy and live birth rates^(2,6,7)^. Under normal conditions, trophoblastic GnRH has been implicated as one of the primary regulators of the synthesis and secretion of hCG in peri-implantation embryos in murine models. Similarly, human placental GnRH plays an important role in the synthesis and secretion of hCG^(11,18)^. Alternatively, GnRHa may also increase pregnancy rates by increasing the synthesis of hCG in the endometrium. However, despite these observations, the precise mechanism by which GnRHa improves pregnancy rates has remained unknown. Finally, *in vitro* studies investigating the effects of GnRHa during cleavage and implantation will be necessary to fully understand the mechanisms on GnRHa activity.

### Study Limitations

The most important limitation of our study is that only a single physician used GnRHa injections, so we cannot rule out the selection or performance bias in the follow-up of IVF treatment. The retrospective nature of this analysis represents the second significant limitation of this work; however, we tried to minimize both limitations by strictly controlling the inclusion and exclusion criteria. Patients with thin endometriums were excluded from the study; although there are studies suggesting the opposite^(19)^, most authors emphasize that pregnancy rates were lower in patients with thin endometriums^(20,21,22)^. Similarly, the exclusion of patients aged >40 years was preferred to limit the effects of embryo quality on implantation. Only first or second IVF cycles of the same patient were included in this analysis to minimize the effects of recurrent implantation failure and to exclude the recurrent number of IVF cycles of the same patient. Finally, only cycles in which grade 1 or 2 embryos were transferred were included as a means of controlling for the effects of embryo quality.

## CONCLUSION

No statistically significant differences in terms of implantation, pregnancy, live birth, and miscarriage rates were observed in patients treated with GnRHa prior to ET relative to the controls. According to this study, GnRHa injection into the uterine cavity prior to ET is not recommended as a means of increasing implantation or pregnancy rates in IVF. However, in order to be able to see the net effect of GnRHa injection into the uterine cavity before ET in IVF, further prospective randomized controlled studies are needed.

## Figures and Tables

**Table 1:**
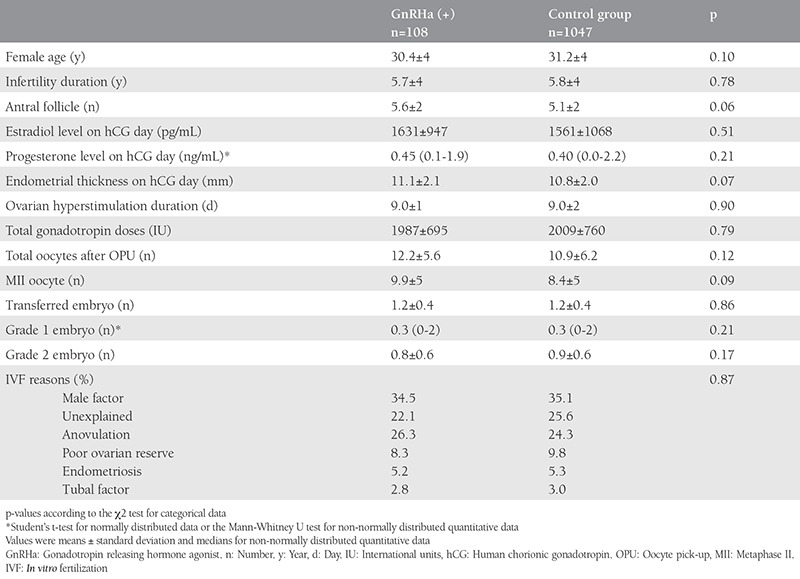
Characteristics of study groups

**Table 2:**
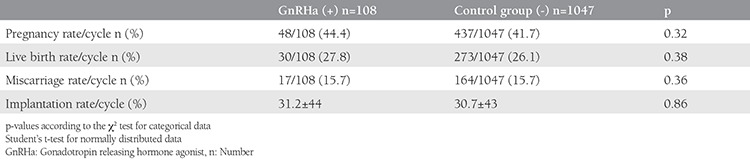
In vitro fertilization outcomes of the two groups

**Table 3:**
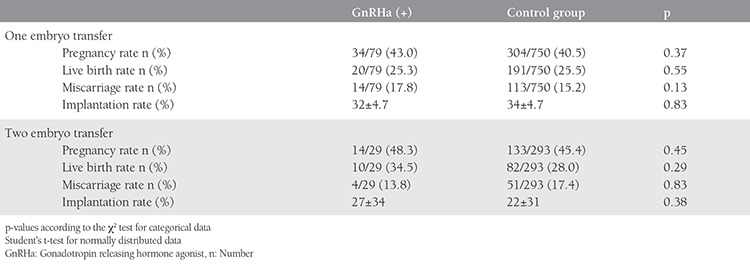
Outcomes of in vitro fertilization according to the number of transferred embryos
